# Secure Inference on Homomorphically Encrypted Genotype Data with Encrypted Linear Models

**DOI:** 10.21203/rs.3.rs-2722738/v1

**Published:** 2023-04-03

**Authors:** Meng Zou, Guangyang Zhang, Fan Zhang, Guoping Liu

**Affiliations:** Huazhong University of Science and Technology; Huazhong University of Science and Technology; Tencent; Huazhong University of Science and Technology

**Keywords:** Homomorphically Encryption, Genotype to Phenotype, CKKS, iDASH-2022

## Abstract

**Background:**

Accurate models are crucial to estimate the phenotypes from high throughput genomic data. While the genetic and phenotypic data are sensitive, secure models are essential to protect the private information. Therefore, construct an accurate and secure model is significant in secure inference of phenotypes.

**Methods:**

We propose a secure inference protocol on homomorphically encrypted genotype data with encrypted linear models. Firstly, scale the genotype data by feature importance with Xgboost or Adaboost then train linear models to predict the phenotypes in plaintext. Secondly, encrypt the model parameters and test data with CKKS scheme for secure inference. Thirdly, predict the phenotypes under CKKS homomorphically encryption computation. Finally, decrypt the encrypted predictions by client to compute the 1-NRMSE/AUC for model evaluation.

**Results:**

5 phenotypes of 3000 samples with 20390 variants are used to validate the performance of the secure inference protocol. The protocol achieves 0.9548, 0.9639, 0.9673 (1-NRMSE) for 3 continuous phenotypes and 0.9943, 0.99290 (AUC) for 2 category phenotypes in test data. Moreover, the protocol shows robust in 100 times of random sampling. Furthermore, the protocol achieves 0.9725 (the average accuracy) in an encrypted test set with 198 samples, and it only takes 4.32s for the overall inference. These help the protocol rank top one in the iDASH-2022 track2 challenge.

**Conclusion:**

We propose an accurate and secure protocol to predict the phenotype from genotype and it takes seconds to obtain hundreds of predictions for all phenotypes.

## Introduction

The research on genotype-to-phenotype are crucial to uncover the gene functions and the mechanisms in distinct phenotypic outcomes [1]. The high throughput genomic data makes it may be possible to predict the phenotype from genotype. While the inference of genotype to phenotype is a complex problem due to the intricate factors such as genotypes, epigenetic variants and their interactions [2]. Moreover, an individual of same genotype may develop to thousands of different diseases, which makes it still a huge challenge in achieving accurate predictions for these phenotypes efficiently[3]. Because of the sensitive nature of genotype and phenotype data, a secure and accurate model is essential for the secure inference of the predictions.

Linear or logistic regression models are generally applied in Genome-wide association studies (GWASs) such as SNPTEST and PLINK [4–6]. However, the linear models may be overfitting because of the number of genotypes far exceed phenotypic outcomes. Regularized linear regression models such as ridge regression, lasso, elastic net and their extensions could overcome the overfitting problems and select a functional genotype set for phenotype estimations [7–9]. While linear models could only capture additive effects, ensemble-based machining learning methods such as Xgboost or Adaboost could select the epistasis genotypes as well and may achieve better performance [10]. Either linear or non-linear models assist to construct an accurate model to infer the phenotype from genotype.

The sensitive nature of genotype and phenotype data urges to develop the secure inference models for phenotype prediction. Besides, the track 2 of iDASH-2022 appeals to develop a secure model evaluation on homomorphic encrypted genotype data via protecting both model parameters and genotypes. Homomorphic encryption (HE) is a cryptosystem that enables homomorphic operations on encrypted data and is considered as one of the most important primitives for privacy-preserving applications. Most of the current HE schemes can be categorized into word-wise HE (such as BFV [11], BGV [12] and CKKS [13]) and bit-wise HE (such as FHEW [14] and TFHE [15]). Among these schemes, Cheon-Kim-Kim-Song (CKKS) is regarded as the unique scheme to support homomorphic operations on float/complex number naturally. Therefore, CKKS could be utilized to construct a secure inference of phenotype from genotype.

To make the secure inference efficiently, we propose an accurate and secure inference protocol on homomorphic encrypted genotype data with encrypted linear models. Firstly, scale the genotype data by feature importance with Xgboost or Adaboost then train a linear model to predict the phenotypes in plaintext. Secondly, encrypt the model parameters and genotype data with CKKS for secure inference. Thirdly, predict the phenotypes under CKKS homomorphic encryption. Finally, decrypt the encrypted predictions by client to compute the 1-NRMSE/AUC for model evaluation.

## Methods

### Overview of the secure inference protocol

**Figure 1:**
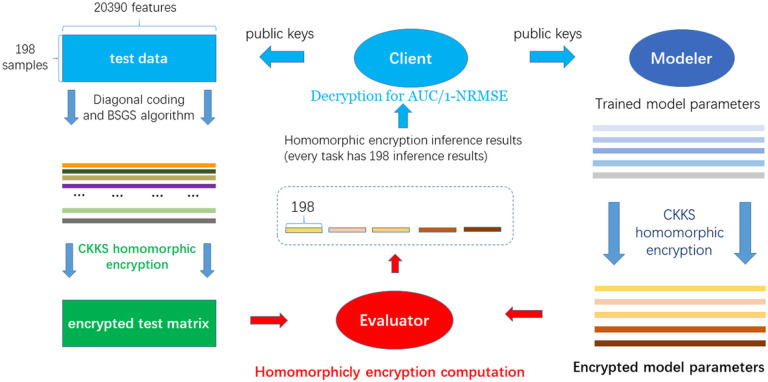
The flowchart of the secure inference protocol. **Step1.** Client generates private key and public key and broadcasts the public keys to Modeler and Evaluator; **Step 2.** Client encrypts test data with the public key by diagonal coding and BSGS algorithm and CKKS homomorphic encryption then sends the encrypted results to Evaluator; **Step 3.**Modeler encrypts model parameters with received public key by CKKS homomorphic encryption then sends the encrypted results to Evaluator; **Step 4.** Evaluator executes homomorphically secure model inferences and sends the encrypted predictions to Client; **Step 5.** Client decrypts the ciphertext of predictions then the decrypted predictions are used for computing 1-NRMSE and AUC.

The secure inference consists of three parties: Client, Modeler, and Evaluator. 198 samples with 20390 features/variants are taken as an example to illustrate the details (Fig. 1). Firstly, Client generates private key and public key (for encryption), relinearization keys (for ciphertext multiplication) and Galois keys (for ciphertext rotation), and broadcasts these public keys to Modeler and Evaluator; Secondly, Client encrypts test data with the public key by diagonal coding, BSGS algorithm and CKKS homomorphic encryption and sends the encrypted results to Evaluator; Thirdly, Modeler encrypts model parameters with received public key and sends the encrypted results to Evaluator; Fourthly, after receiving the encrypted test data matrix and encrypted model parameters, Evaluator executes homomorphically secure model inferences with received relinerization keys and galois keys, and sends the encrypted predictions to Client; Finally, Client decrypts the ciphertext of predictions then the decrypted predictions are used for computing 1 − NRMSE and AUC.

### Linear models with feature importance for predictions of phenotype from genotype in plaintext

*X*∈ R^*m*×*n*^ is the genotype matrix and *X*_*ij*_ denotes the *j*-th variant for *i*-th sample. *Y* ∈ *R*^*m*×*K*^ is the phenotype matrix and *Y*_*ik*_ denotes the *k*-th phenotype for *i*-th sample. Xgboost or Adaboost is used to obtain the feature importance for each phenotype then scale the raw genotype matrix by

$$X^{\left(k\right)}=X*\text{diag}\left(F_{k}\right)$$

Where *F*_*k*_ is the feature importance for the *k*-th phenotype.

If *Y*_*k*_ is the continuous phenotype, then the linear regression model should be

$$Y_{k}=X^{\left(k\right)}\times M_{k}+w_{0k}+\epsilon_{k}$$

Where *M*_*k*_ is the linear regression parameter and *w*_0*k*_ is the intercept term. Let

$$W_{k}=M_{k}\times \text{diag}\left(F_{k}\right)$$

Then the final model is

$$Y_{k}=X\times W_{k}+w_{0k}$$

If *Y*_*k*_ is the category phenotype (i.e. 0 and 1), then the logistic regression model should be

$$p_{k}=\frac{1}{1+\text{exp}\left(X^{\left(k\right)}\times M_{k}+w_{0k}\right)}$$

Where *p*_*k*_ is the probability of predicting the phenotype to be 1.

Similarity, the final model could be

$$p_{k}=\frac{1}{1+\text{exp}\left(X\times W_{k}+W_{0k}\right)}$$

In summary, both final models adopt the Linear Model with Feature Importance (LMFI) for phenotype inference.

### CKKS Scheme

For a 2-power number N, we write *R*_*N*_ = Z[*X*]/ (*X*^*N*^ + 1) and *R*_*N*,*q*_ = *R*_*N*_*/qR*_*N*_ ≡ *Z*_*q*_[*X*]/(*X*^*N*^+ 1). The lower-case letters with a “hat”symbol such as $\hat{a}$ represents some element in *R*_*n*_, and *a*_*j*_ is denoted as the *j*-th coefficient of $\hat{a}$. The dot symbol · such as $\hat{a}\cdot \hat{b}$ is denoted as the multiplication of ring elements. We use bold lower-case letters symbol such as *a* to represent vectors, *a*[*j*] to represent the *j*-th component of *a*, and *a*∥*b* to represent the concatenation of vectors. Denote by *a* ≪ *k* the left-hand-side rotation of the vector components. Denote by *a*^*T*^*b* the inner product of vectors and *a* ∘ *b* the Hadamard product of vectors, i.e., the element-wise multiplication. We use bold upper-case letters such as *M*to denote matrices, and *M*[*i*, *j*] to denote the (*i*, *j*) entry of *M*.

As Z[X]/ (*X*^*N*^+ 1)is isomorphic to *C*^*N*/2^, the ring structure allows us to encode a real vector *v* ∈ *R*^*l*^ as a ring element of *R*_*N*,*q*_ with *l* ≤ *N*/2. The addition/multiplication in *R*_*n*_ corresponds to elementaddition/multiplication of the real(complex) vector *v* ∈ *R*^*l*^. Denote by *Encode*(*v*, *Δ*) ∈ *R*_*N*,*q*_ and $\text{Decode}(\hat{v},\Delta ,l)\in {\text{R}}^{l}$ the encoding of *v* with a scaling factorΔ > 0, and the decoding of $\hat{v}$ with a scaling factor Δ > 0 and a length *l* > 0 respectively.

The Ring Learning With Errors (RLWE) distribution RLWE_*s*_(*N*, *q*, *χ*) with secret *s* ∈ *R*_*N*_ and error distribution *χ* over *R*_*N*_, produces pairs (*a*, *b*) ∈ *R*_*N*_,_*q*_ where *a* ← *R*_*N*_,_*q*_ is chosen uniformly at random, and *b* = *s* · *a* + *e* for *e* ← *χ*. The decisional Ring LWE assumption over *R*_*N*_ with error distribution *χ*, secret distribution *χ*′ and *m* samples, states that when *s* ← *χ*′, the product distribution RLWE_*s*_(*N*, *q*, *χ*)^*m*^ is psedudorandom, i.e., it is computationally indistinguishable from the uniform distribution over (*R*_*N*,*q*_ × *R*_*N*,*q*_)^*m*^. As usual, *χ*′ is the uniform distribution over *R*_*N*,3_ = *R*_*N*_/3*R*_*N*_ and *χ* is the discrete Gaussian distribution.

The security of CKKS scheme is based on RLWE Assumption. The following is the details of CKKS.

1. The key generation algorithm picks *s* ← *χ*′, *e* ← *χ*, *a* ← *R*_*N*,*q*_, and outputs secret key $sk=(-s,1)\in R_{N,q}^{2}$, and public key $pk=(a,b)\in R_{N,q}^{2}$, where *b* = *s* · *a* + *e* follows the RLWE distribution.
2. The encryption algorithm, ${\text{Enc}}_{pk}(\hat{V})$ picks random *u* ← {0, 1}^*N*^ and e = (*e*_0_, *e*_1_) ← *χ*^2^, and outputs $ct=u\cdot pk+e+(0,\hat{v})\in R_{q,N}$, where $\hat{v}=\text{Encode}(v,\Delta )$
3. The approximate decryption algorithm *Dec*_*sk*_(*ct*) outputs $v=\text{Decode}(\hat{v},\Delta )$ where $\hat{v}=\langle sk,ct\rangle \text{modq}$.

By linearity of Enc, CKKS directly supports (bounded) addition of ciphertexts: if *ct*_0_ = (*a*_0_, *b*_0_) and *ct*_1_ = (*a*_1_, *b*_1_) are encryptions of *v*_0_ and *v*_1_ respectively, then the vector sum *ct*_0_ + *ct*_1_ = (*a*_0_ + *a*_1_, *b*_0_ + *b*_1_) mod*q* is an encryption of *v*_0_ + *v*_1_. The plaintext-ciphertext multiplication is ${\hat{v}}_{0}\cdot ct_{1}=\left({\hat{v}}_{0}\cdot a_{1},{\hat{v}}_{0}\cdot b_{1}\right)\text{mod}q$ is an encryption of *v*_0_ ∘ *v*_1_, where ${\hat{v}}_{0}=\text{Encode}(v,\Delta )$.

For homomorphic multiplication/rotation, extra public keys are needed. Denote by *EK*/*RotK* the evaluation key for homomorphic multiplication/rotation, respectively.

#### Slot-wise Multiplication.

Using EK, the product of two ciphertexts *ct*_0_ = (*a*_0_, *b*_0_), *ct*_1_ = (*a*_1_, *b*_1_) is computed as *Mul*(*ct*_0_, *ct*_1_;*EK*) = *ct*_0_ × *ct*_1_ = (*a*_0_*b*_0_ + *a*_1_*b*_0_, *b*_0_*b*_1_) + *ReLin* (*a*_0_*a*_1_; *EK*), where *ReLin* (*α*; *EK*) = (*α*_0_, *α*_1_) such that *α*_0_ + *α*_1_ · *sk*= *α* · *sk*^2^ + *e* for some error *e*.

#### Rotation.

Given the ciphertext ct which encrypts *Encode*(v, Δ), an integer *k* ∈ *N*, and a rotation key *RotK*, the operation *RotL*^*k*^(*ct*; *RotK*) results in an CKKS ciphertext that encrypts the left-hand-side rotated vector Encode(*v* ≪ *k*, Δ).

#### Rescale.

Given the CKKS ciphertext ct which encrypts *Encode*(*v*, Δ), and a factor Δ′ ∈ R, the operation *Rescale*(*ct*, Δ′) results in a ciphertext (with a smaller modulus) that encrypts *Encode*(*v*, Δ/Δ′).

#### Self-repeating.

*Decode*(*Encode*(*v*∥ ⋯ ∥ *v*, *Δ*), *Δ*, *l*) = *v*. In other words, the encoding of some self-repeating vectors can be viewed as the encoding of a single copy.

### Homomorphic Linear Evaluation

There are some existing approaches to homomorphic linear evaluation, i.e., the homomorphic multiplication of plain matrix and encrypted vector [16–18]. The method proposed by Wenjie Lu et al. could cover both “tall” and “short” matrices efficiently and it is proper for our solution[18]. The following is the details of the matrix encoding and the process of homomorphic linear evaluation.

### Algorithm 1. Matrix Encoding

#### Input

A plain matrix with and a scaling factor .

#### Output

elements as encoded matrix.

1. Tiling and Diagonals. Define *l* vectors ${\left\{m_{j}\right\}}_{j=0}^{l-1}$ by going through the rows and columns of *M*
$$m_{j}[r]=M[r\text{mod}l,r+j\text{mod}n]\text{for}\;r\in \{0,1,\cdots n\}$$
2. Let $g=\left\lceil \sqrt{l}\right\rceil$ and b= ⌈*l*/g⌉, compute ${\hat{m}}_{i}=\text{Encode}\left(m_{i_{1}g+i_{2}}\gg i_{1}g,\Delta \right)$ for *i*_1_ ∈ {0, 1, ⋯, *b* − 1} and *i*_2_ ∈ {0,1, ⋯, *g* − 1}.
3. Output ${\left\{{\hat{m}}_{i}\right\}}_{i=0}^{l-1}$.

### Algorithm 2. Homomorphic Linear Evaluation

#### Input

with being encrypted. Rotation key Encoded matrix of, which the column size is. The scaling factor used to encode is.

#### Output

ciphertext with encrypted.

1. Let $g=\left\lceil \sqrt{I}\right\rceil$. For *i*_2_ ∈ {0, 1, ⋯, g − 1}, compute $c_{i_{2}}={\text{RotL}}^{i_{2}}\left(ct_{z};\text{RotK}\right)$.
2. Let *b*= ⌈*l*/g⌉. Compute $ct=\sum_{i_{1}=0}^{b-1}{\text{RotL}}^{i_{1}g}\left(\sum_{i_{2}=0}^{g-1}{\hat{m}}_{i_{1}g+i_{2}}\cdot c_{i_{2}}\right)$.
3. Let *γ* = log(*n*/*l*) and *ct*_0_ = *ct*. Update iteratively for1 ≤ *j*≤ *γ*
$$ct_{j}={\text{RotL}}^{l2^{j}}\left(ct_{j-1}\right)+ct_{j-1}.$$
4. Output *Rescale*(*ct*_γ_, Δ) as *ct*_*out*_.

The rectangular matrix *M* is converted to a square matrix by repeating the rectangular matrix itself (called tiling) instead of expanding the rows (resp. cols) of *M* with zero-padding to be squared. A subset of the diagonals of the tiling matrix are constructed in Step 1 by looping through the rows and columns of *M*. This tiling is always possible without zero-padding because the number of rows and columns of *M* is always a power-of-2 value. The baby-step-giant-step (BSGS technique [19] in Step 2 of Algorithm 1 and Algorithm 2 aims to sum up some products of plaintext-ciphertext with a specific offset of homomorphic rotations. Specifically, $\sum_{j=0}^{l-1}\left(m_{j}\circ z\ll j\right)=\sum_{i_{1}=0}^{b-1}\left(\sum_{i_{2}=0}^{g-1}\left(\left(m_{i_{1}g+i_{2}}\gg i_{1}g\right)\circ \left(z\ll i_{2}\right)\right)\right)\ll i_{1}g$. The term $m_{i_{1}g+i_{2}}\gg i_{1}g$ is executed before Encode in CKKS since the cost of homomorphic rotations on encrypted *z* is expensive.

In Step 2 of Algorithm 2. *ct* can be viewed as a ciphertext, which corresponds to the sum of the *l* column vectors (each of size *n/l*), i.e., the matrix multiplication of each *n/l* columns of *M* and *z*. The step 3 of Algorithm 2 aims to sum up the encrypted columns, resulting in *ct*_*γ*_ that encrypts a self-repeating vector *Encode*(*Mz*∥ ⋯ ∥ *Mz*, Δ _*r*_Δ). It can just be viewed as *Encode*(*Mz*, Δ _*r*_Δ) according to the property of the encoding function. Finally, the *Rescale*(· , Δ) is used to reach the same scaling factor of *ct*_*z*_.

The overall computation consists of *l* homomorphic multiplication and *g* + *b* + log*n/l* homomorphic rotations.

### Modification and Optimization

For *l* and *n* not being power-of-2, we naturally extends the rows and columns of *M* with zero padding to fit the matrix encoding requirement. The expanded matrix has both the row size and the column size of being power-of-2.

For *n* larger than *N*/2, we split *M* into distinct submatrix (*M*_1_, *M*_2_, ⋯, *M*_*s*_), where *M*_1_ ∥ *M*_2_ ∥ ⋯ ∥*M*_s_ = *M* and the column size of each submatrix is no more than *N*/2. Therefore, the encoding of *M* is converted into the encoding of *s* submatrix, which implies *s* homomorphic linear evaluations. According to the linearity of homomorphic rotation, homomorphic multiplications in each homomorphic linear evaluation are firstly executed and summed together followed by the execution of homomorphic rotations, The number of executing homomorphic rotations is reduced from *s* to only one. That is to say, the cost of homomorphic rotations are independent of the number of submatrices.

Assume only homomorphic linear evaluation is needed, the encoding of *M* can be improved by regarded two adjacent float rows as one complex row. That is, the same components of first row and second row are the real part and imaginary part of complex row respectively. With the improvement, the length of $\left\{{\hat{m}}_{j}\right\}$ is reduced by nearly half. After homomorphic linear evaluation, the ciphertext is the encryption of the complex vector which the real part and imaginary part of each component is the adjacent component of *Mz*. With the modification of matrix encoding, the time complexity and space complexity of homomorphic linear evaluation are almost reduced by half.

For homomorphic linear evaluation of encrypted matrix and encrypted vectors, the matrix is encrypted with ${\left\{{\hat{m}}_{i}\right\}}_{i=0}^{l-1}$, which are the outputs of Algorithm 1. In Step 2 of Algorithm 2, $\sum_{i_{2}=0}^{g-1}{\hat{m}}_{i_{1}g+i_{2}}\cdot c_{i_{2}}$ is replaced by $\sum_{i_{2}=0}^{g-1}\text{Mul}\left(ct_{{\hat{m}}_{i_{1}g+i_{2}}},c_{i_{2}};EK\right)$, where $ct_{{\hat{m}}_{i_{1}g+i_{2}}}$ is the ciphertext of encrypted ${\hat{m}}_{i_{1}g+i_{2}}$. Since the cost of *Relin*(· ; *EK*) is expensive, We can sum up multiple ciphertext before *Relin* in the case of *n* > *N*/2, i.e. multiple homomorphic linear evaluation of encrypted submatrix and encrypted sub-vector and ciphertext summation. That is to say, if we need to compute multiple-multiplication-andsummation of between multiple *ct*_0,*i*_ = (*a*_0,*i*_, *b*_0,*i*_) and *ct*_1,*i*_ = (*a*_1,*i*_, *b*_1,*i*_), then (*a*_0_,_i_*b*_0_,_I_ + *a*_1_,_i_*b*_0_,_i_, *b*_0_,_i_*b*_1_,_*i*_) and *a*_0_,_*i*_*a*_1_,_*i*_ are firstly computed and summed up. As input, ∑ _*i*_*a*_0_,_*i*_*a*_1_,_*i*_ are executed by *Relin* with *EK* secondly. Finally, the ∑_*i*_(*a*_0_,_*i*_*b*_0_,_*I*_ + *a*_1_,_*i*_*b*_0_,_*i*_, *b*_0_,_*i*_*b*_1_,_*i*_) and Relin(∑_*i*_*a*_0,*i*_*a*_1,*i*_;*EK*) are summed up, which is equivalent to ∑_*i*_*Mul*(*ct*_0,*i*_, *ct*_1,*i*_). Note that *Relin* is executed only once, and the cost of expensive *Relin* is independent of the number of ciphertext pairs ((*ct*_*i*,0_, *ct*_*i*,1_)).

### Evaluation

The performance of the secure inference protocol is evaluated by the model accuracy and the running time. Specifically, the model accuracy is achieved by 1-NRMSE for continuous phenotype and AUC for category phenotype. Here NRMSE of the k-th phenotype is calculated by

$${\text{NRMSE}}_{\text{k}}=\frac{\sqrt{\sum_{i=1}^{m}{\left(Y_{ik}-{\hat{Y}}_{ik}\right)}^{2}/m}}{\text{max}\left\{Y_{k}\right\}-\text{min}\left\{Y_{k}\right\}}$$

Where ${\hat{Y}}_{ik}$ is the prediction of the k-th phenotype for sample *i*. Let

$$S_{k}=\{\begin{array}{c} 1-{\text{NRMSE}}_{\text{k}}Y_{k}\text{iscontinous}\\ {\text{AUC}}_{k}\text{Otherwise} \end{array}$$

Then the average model accuracy could be inferred by

$$\bar{S}=\frac{1}{K}\sum_{k=1}^{K}S_{k}$$

Both the average accuracy and running time are adopted to evaluate the performance of the secure inference protocol in the iDASH-2022 track2 challenge.

### Datasets

We validate the secure inference protocol in a genotype dataset containing 3000 samples with 20390 variants. Each variant typically contains three genotypes such as AA, Aa and aa. Besides, all the samples contain 5 phenotypes, where three are continuous phenotypes and two are category phenotypes (i.e. 0 and 1). Furthermore, 198 samples are remaining for the blind test of the secure inference protocol. All these data are provided by the iDASH-2022 track2 http://www.humangenomeprivacy.org/2022/.

## Results

### LMFI outperform other methods in plaintext

**Figure 2:**
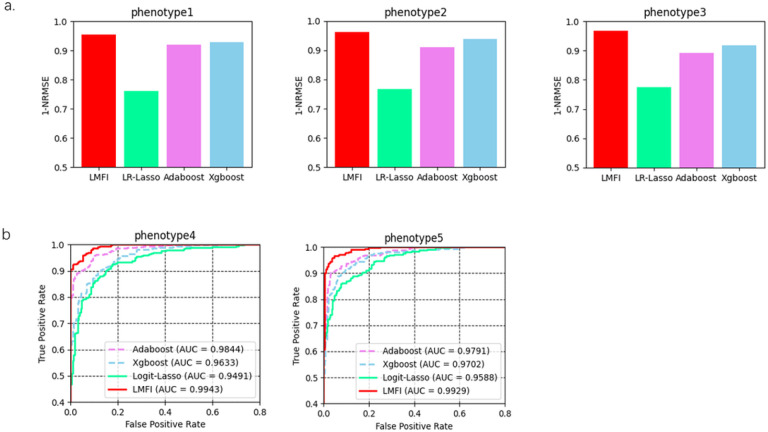
The comparisons of different methods. The performance of LMFI, Xgboost, Adboost, LR/Logit-lasso on continuous phenotypes (a) and category phenotypes (b). 1-NRMSE(Normalized Root Mean Square Error) is used to evaluate the continous phenotypes and AUC is used to evaluate the category phenotypes. LMFI shows the best perfomance among these methods.

To demonstrate the performance of LMFI in the inference of phenotype from genotype, we applied it to the dataset containing 5 phenotypes of 3000 samples with 20390 variants, and ten percent of the dataset is randomly selected as test data. LMFI achieves 0.9548, 0.9639, 0.9673, 0.9943, 0.9929 for overall phenotypes, respectively (Fig. 2). which performs much better than linear models with lasso in both continuous and category phenotypes. Furthermore, LMFI also shows better than non-linear models, such as Adaboost achieves 0.9185, 0.9151, 0.8936, 0.9844, 0.9791 and Xgboost achieves 0.9291, 0.9395, 0.9180, 0.9633, 0.9702.

### The secure inference protocol is robust

**Figure 3:**
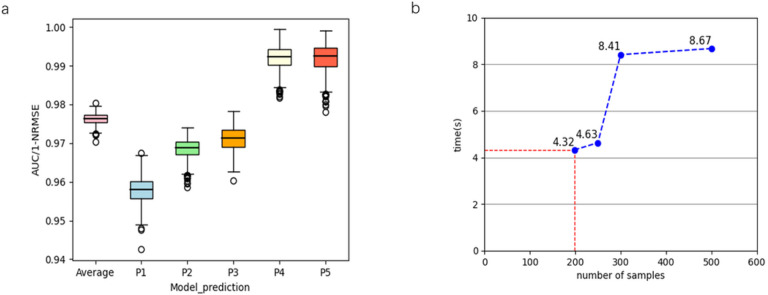
The performance of the secure inference protocol. **a.** The accuracy of the secure inference protocol in100 times of random sampling. **b.** The running time of the secure inference protocol.

To demonstrate the robust of the secure inference protocol, we applied it to a random sampling with 300 test samples for 100 times, and evaluated the performance by mean and standard deviation. The secure protocol could obtain 0.9577 ± 0.0033 (mean and standard deviation), 0.9686 ± 0.0025, 0.9711 ± 0.0030, 0.9921 ± 0.0029, 0.9920 ± 0.0034 for 5 phenotypes, which indicating that the secure protocol is robust in different experiments (Fig. 3a). Furthermore, the average of the secure protocol achieves 0.9725 in a blind test with 198 samples.

### The secure inference protocol is efficient

To demonstrate the efficient performance of the secure protocol, we test the performance with different sample size. Here we choose *N* = 8192, the coeff modulus is the multiplication of three primes with bit size 60, 60, 60 respectively by obeying the homomorphic encryption white paper [20]. For 198 × 20390 test data samples, which are regarded as 5 submatrix (with first four 198 × 4096 matrices and last 198 × 4006 matrix), we extend the five matrices to be 256 × 4096 matrices with zero padding. By assembling the adjacent rows of each submatrix into 128 × 4096 complex submatrix, we encode the complex submatrices to obtain 5 × 128 elements in *R*_*N*_. In the process of homomorphic linear evaluation, 5 × 128 homomorphic multiplications and 16 + 8 + 5 homomorphic rotations are needed.

The protocol only takes 4.32s for 198 samples (Fig. 3b). With the sample size arise, it does not change much (4.63s for 250 samples). Even with 500 samples, it takes 8.67s to obtain the final predications. All the computation are compiled by AMD EPYC 7K64@2.6GHz, running with 4 processes and the memory is 8GB.

## Discussion And Conclusion

Construct an accurate, secure and efficient phenotype prediction model is essential for privacy and security computation. We have developed a secure inference protocol with encrypted linear models and it achieves good performance in the inference of phenotype and shows robust in 100 times of random sampling. Besides, it is very efficient and only takes seconds to predict hundreds of samples. However, the protocol also needs to be further developed. Firstly, the protocol has not been test on more datasets. Secondly, the protocol should be further improved in homomorphic encrypted computation. Thirdly, the linear models could be extended to non-linear models to achieve better accuracy and the homomorphic encrypted computation methods should be corresponded to be transformed. In conclusion, we have developed an accurate, secure and efficient phenotype prediction protocol and it takes only seconds to predict hundreds of samples.

## Data Availability

The datasets are available in the http://www.humangenomeprivacy.org/2022/ providing by the iDASH-2022 track2.

## Abbreviations

iDASH: integrating Data for Analysis, anonymization, and SHaring
LMFI: Linear Model with Feature Importance
NRMSE: Normalized Root Mean Square Error
AUC: Area Under Curve
HE: Homomorphic Encryption
RLWE: Ring Learning With Errors
BSGS: Baby-Step-Giant-Step
CKKS: Cheon-Kim-Kim-Song
